# Malignant Pustule

**Published:** 1886-05-20

**Authors:** F. W. Moffitt

**Affiliations:** Randolph, Wis.


### MALIGNANT PUSTULE.

By F. W. MOFFITT, M. D., RANDOLPH, WIS.

On the 26th of May, 1885, a six-year old daughter of H. Z——, German, came into dinner with a slight scratch on the left side of her nose. She had been playing with some chickens, dead from chicken cholera. She complained of some pain in a short time, and in a few hours a pustule had formed, containing dirty, yellow pus. In a few more hours the pus had become clouded with dark colored masses, and some ædema had set in. By morning of the

27th the ædema involved the eyelids and whole upper part of face; the pustule had a livid, indurated border, and there was some fever. The parents being averse to running a doctor bill, treated it with local sour-milk applications, and internally some herb tea. The case, of course, continued to grow worse, and on the morning of the 29th I was called in.

The history, as near as I could learn, was as I have given above. I found in the seat of the pustule a black crust surrounded by a livid, raised, indurated area three or four lines thick. The ædema had closed both eyes so completely that I could scarcely open them, and had extended into the frontal region nearly to the coronal suture, near which another pustule was forming, and another had formed over the right orbit, about an inch above it. When the crust was removed from the original sore, it revealed an ulcer with a sloping floor, perforated at the bottom by an orifice about the size of a pin hole. By pressing upon the indurated base this orifice gave exit to a bloody pus. There was a general depressed state of the system with a tendency to coma. The urine was acid, high colored and scanty. Evacuations semi-solid and possessing a terrible stench.

From the history and present symptoms I pronounced the case malignant pustule. Excised the ulcers and cauterized. Iron, quinine, and stimulants with carbolized water dressings. Also freely opened a sinus connecting first two pustules.

Prognosis unfavorable, because I thought the pus had already entered the ethmoidal sinuses; hence the head symptoms.

May 30.—Case appeared somewhat improved; ædema much less; pulse slower and stronger, but coma still impending.

May 31.—Considerable stupor, muscular twitchings, nervous startings; urine retained, and when voided was done so involuntarily, as also were the feces.

From this the case passed very rapidly on a downward course, which resulted in death on the 2d of June.—*Peoria Med. Monthly.*

